# Supplementary material for: Anhedonic Traits Do Not Impair Performance in a 3-Arm Bandit Task
Source: Comput Psychiatr. 2026 Apr 13;10(1):58–84. doi: 10.5334/cpsy.135 (PMC13089363; doi:10.5334/cpsy.135)
Supplement: Supplementary Material. — Supplementary material containing additional reaction-time (RT) analyses and robustness checks for the reinforcement-learning models, including subjectlevel and trial-level RT comparisons between anhedonic and non-anhedonic groups, and posterior group-difference HDI summaries and density plots for lapse-augmented model variants. [file cpsy-10-1-135-s1.pdf]

**Supplementary Material**

Reaction Time Group Comparisons Calculation:

**S1. Reaction Time (RT) Analysis**

Reaction times were computed as the duration between the onset of the response screen and the participant's recorded keypress on each trial. Trials with missed responses or implausibly fast (<200 ms) or slow (>3000 ms) RTs were excluded. RTs were averaged across all valid trials per participant. We then compared mean RTs between the Anhedonic and Non-Anhedonic groups using an independent samples t-test. RT distributions were visualized with scatter plots and violin plots, and no significant group differences were observed.

**Figure S1. Group Comparison of Reaction Times**

Mean reaction times (RTs) across 200 trials are plotted for the Anhedonic (n = 111) and Non-Anhedonic (n = 95) groups. Shaded regions represent the standard error of the mean (SEM) at each trial. Both groups exhibited a rapid decrease in RTs over early trials followed by stabilization, reflecting task adaptation. Although the Anhedonic group showed numerically faster RTs across trials, this difference was not statistically significant (t (203.61) = –1.98, p = 0.050, Cohen’s d = –0.28). RTs were computed as the latency from stimulus onset to participant keypress. Trials with implausible RTs (<200 ms or >3000 ms) or missed responses were excluded from the analysis. These results suggest that differences in reaction time are unlikely to explain group effects in learning behaviour or model parameters.

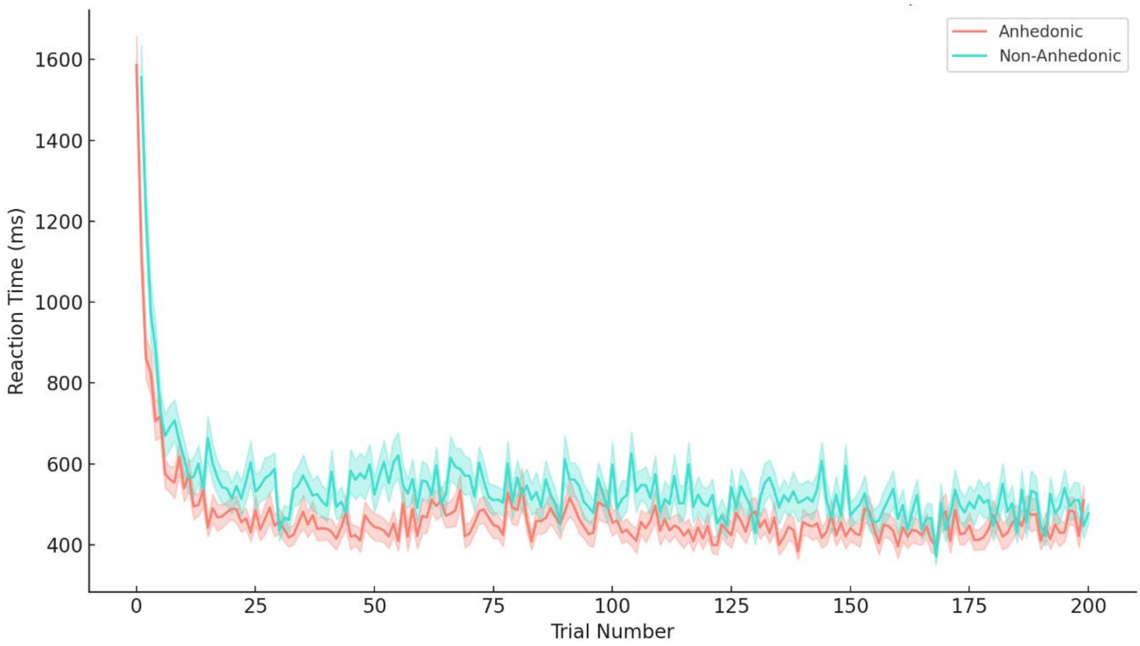


**Supplementary Figure S1. Reaction time across trials for anhedonic and non-anhedonic groups**. Mean reaction time (RT) across trials for anhedonic and non-anhedonic participants

in the 3-arm bandit task. Lines show the mean RT at each trial, and shaded regions show the standard error of the mean (SEM). Both groups show a sharp reduction in RT over the first 5–10 trials followed by a more stable regime. Across most of the task, the non-anhedonic group shows higher mean RTs than the anhedonic group, consistent with the subject-level analyses reported in Supplementary Section 1.

.

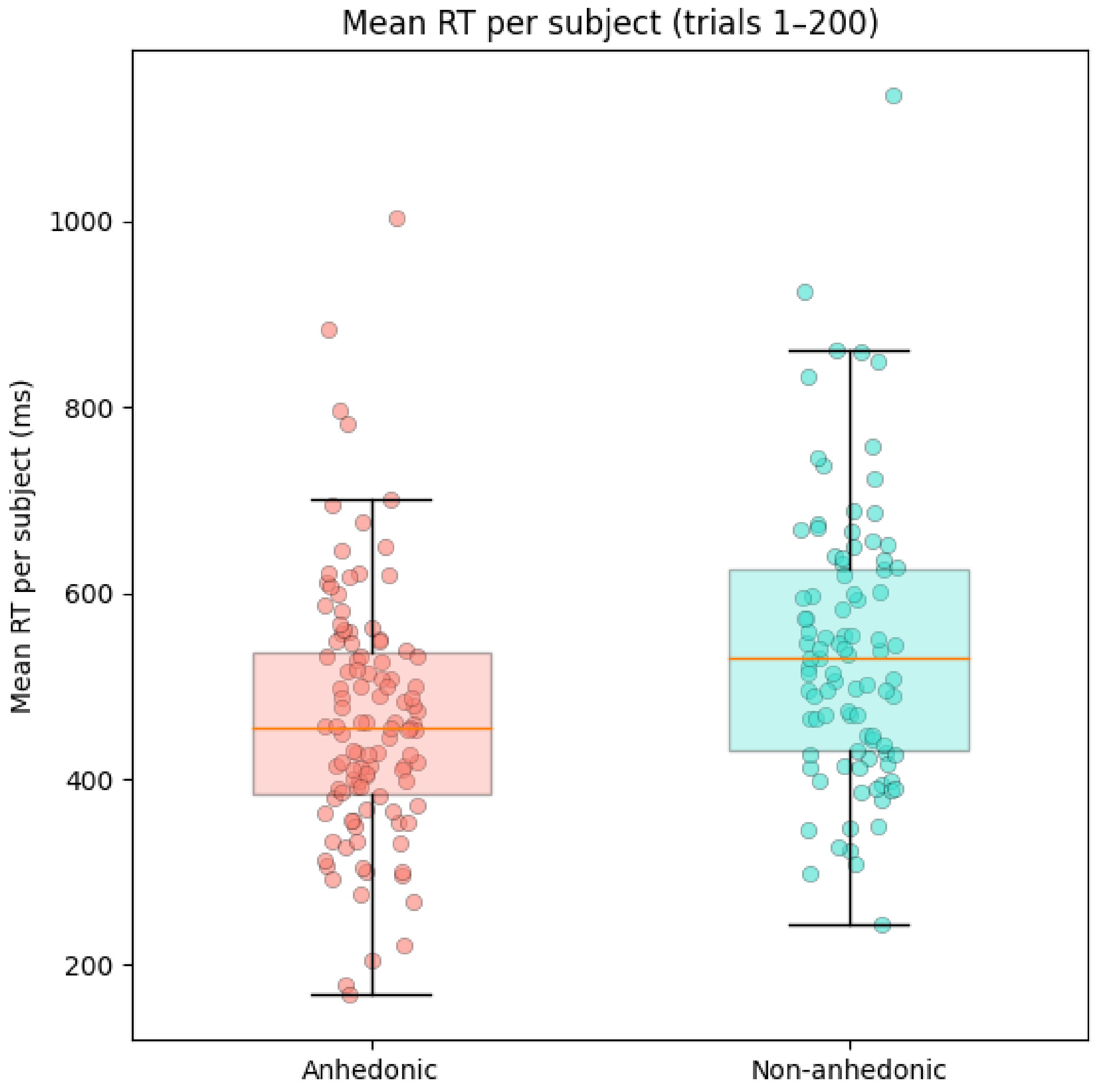


**Supplementary Figure S2. Mean RT per subject across all trials (1–200)**Mean RT per subject across all trials (1–200) for anhedonic and non-anhedonic participants. Each point represents one participant's mean RT. Boxplots show the median and interquartile range for each group (whiskers indicate the range excluding outliers). Non-anhedonic participants are slower on average (Anhedonic: N = 111, M = 465.53 ms, SD = 135.33; Non-anhedonic: N = 95, M = 539.62 ms, SD = 148.72; t(192.03) = –3.71, p = 0.00027).

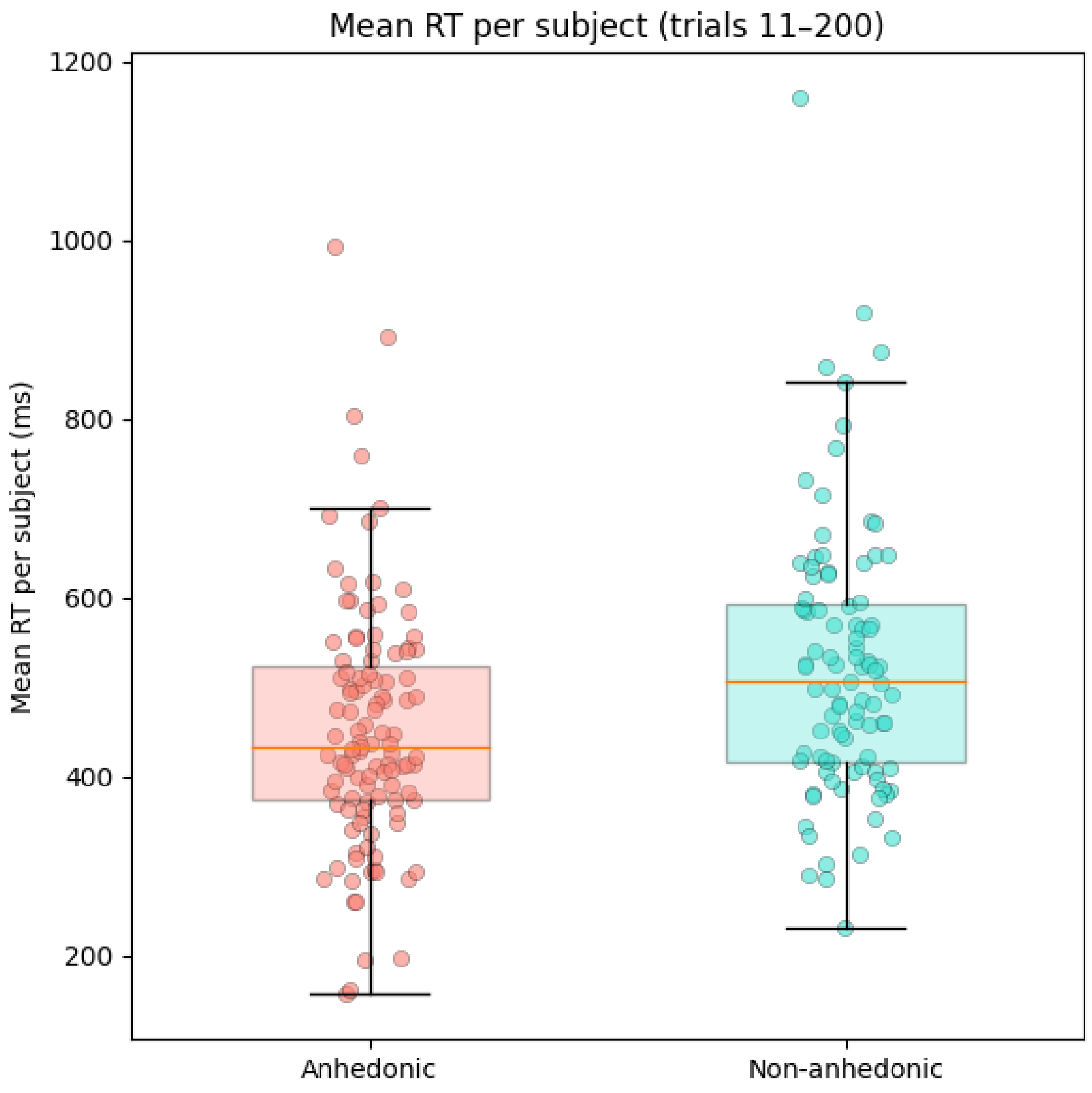


**Supplementary Figure S3. Mean RT per subject in the stable regime (trials 11–200)**Mean RT per subject computed from trials 11–200 only, to focus on the more stable regime after the initial rapid change in RTs. As in Supplementary Figure SRT2, each point represents one participant, overlaid on boxplots showing group medians and interquartile ranges. The slowing in the non-anhedonic group remains evident when early trials are excluded (Anhedonic: M = 453.13 ms, SD = 136.78; Non-anhedonic: M = 524.48 ms, SD = 150.91; t(191.68) = –3.53, p = 0.00052), indicating that the effect is not driven solely by early outlier trials.

**S2. Lapse Models Group HDIs comparisons**

Robustness checks using lapse-augmented models

To confirm that our group-level inferences were not dependent on excluding lapse terms, we estimated two lapse-augmented models separately within the anhedonic and non-anhedonic groups and compared posterior group differences. For the model with separate reward and punishment learning rates and sensitivities plus lapse (banditNarm_lapse), the 95% HDIs for group differences included zero for reward learning rate (HDI [−0.110, 0.054], pd = 0.739), punishment learning rate (HDI [−0.0566, 0.106], pd = 0.737), reward sensitivity (HDI [−1.47, 1.26], pd = 0.641), punishment sensitivity (HDI [−1.45, 0.758], pd = 0.734), and lapse (HDI [−0.00747, 0.0232], pd = 0.802).

We repeated the same analysis for the single learning rate lapse model with separate reward and punishment sensitivities (banditNarm_singleA_lapse). Again, all group-difference 95% HDIs included zero: shared learning rate (HDI [−0.0923, 0.0734], pd = 0.612), reward sensitivity (HDI [−1.68, 1.21], pd = 0.630), punishment sensitivity (HDI [−1.04, 0.692], pd = 0.681), and lapse (HDI [−0.00835, 0.0251], pd = 0.851). Posterior densities are shown in Figures S4 and S5. Together, these robustness checks indicate that the null group findings for learning, sensitivity, and lapse parameters persist when using lapse-augmented models, including those that performed best in model comparison.

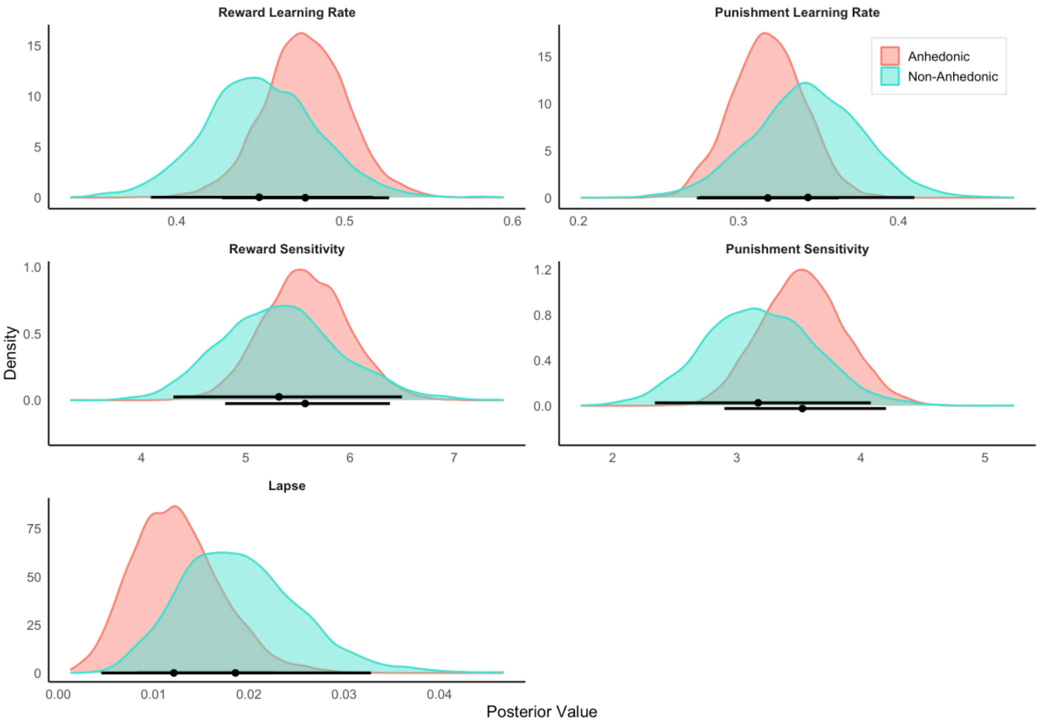


**Supplementary Figure S4. Posterior parameter densities by group for banditNarm_lapse**. Posterior distributions for reward learning rate (Arew), punishment learning rate (Apun), reward sensitivity (R), punishment sensitivity (P), and lapse (ξ), estimated separately in anhedonic and non-anhedonic groups. Black points and horizontal intervals indicate posterior central tendency and 95% HDIs for each group. For all parameters, the 95% HDIs for group differences included zero.

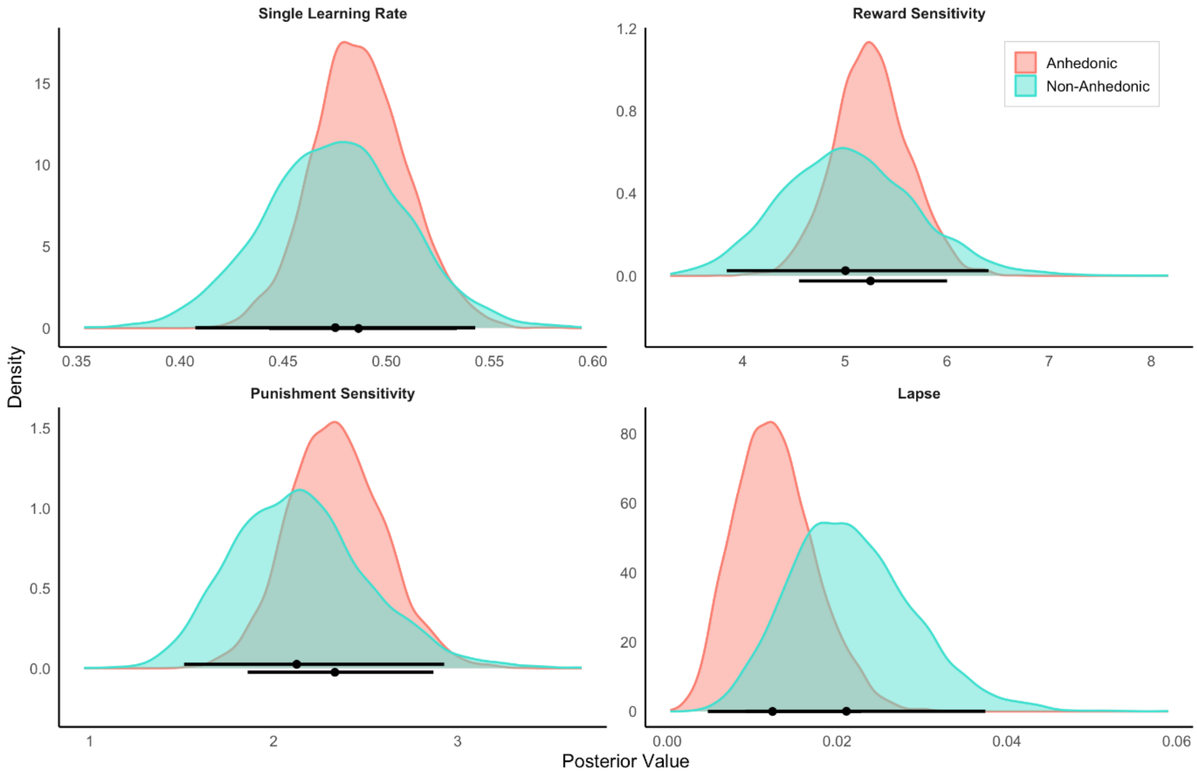


**Supplementary Figure S5. Posterior parameter densities by group for banditNarm_singleA_lapse.** Posterior distributions for shared learning rate (A), reward sensitivity (R), punishment sensitivity (P), and lapse (ξ), estimated separately in anhedonic and non-anhedonic groups. Black points and horizontal intervals indicate posterior central tendency and 95% HDIs for each group. For all parameters, the 95% HDIs for group differences included zero.

**Model variants tested:**

1. https://github.com/CCS-Lab/hBayesDM/blob/develop/commons/stan_files/banditNarm_2par_lapse.stan

2. https://github.com/CCS-Lab/hBayesDM/blob/develop/commons/stan_files/banditNarm_4par.stan

3. https://github.com/CCS-Lab/hBayesDM/blob/develop/commons/stan_files/banditNarm_delta.stan

4. https://github.com/CCS-Lab/hBayesDM/blob/develop/commons/stan_files/banditNarm_kalman_filter.stan

5. https://github.com/CCS-Lab/hBayesDM/blob/develop/commons/stan_files/banditNarm_lapse.stan

6. https://github.com/CCS-Lab/hBayesDM/blob/develop/commons/stan_files/banditNarm_lapse_decay.stan

7. https://github.com/CCS-Lab/hBayesDM/blob/develop/commons/stan_files/banditNarm_singleA_lapse.stan
